# Nonunion of the femoral neck: Possibilities and limitations of the various treatment modalities

**DOI:** 10.4103/0019-5413.38575

**Published:** 2008

**Authors:** Ernst LFB Raaymakers, René K Marti

**Affiliations:** Orthopaedic Department of the Academical Medical Centre, Amsterdam, Netherlands

**Keywords:** Fracture neck femur, nonunion, osteotomy, prosthetic replacement

## Abstract

Nowadays in cases of nonunions of the femoral neck, the surgeon is tempted to perform prosthetic replacement of the hip, more so if there is also evidence of avascular necrosis of the head of femur. This provides rapid pain relief and allows early mobilization. However, long-term results of hip arthroplasties, especially in younger people and in the presence of osteopenia, are not always as expected; and a less radical approach is worth considering. The intertrochanteric valgization osteotomy, described by Pauwels, is an excellent alternative for healthy patients up to 65 years of age with a nonunion of the femoral neck. A union rate of 80-90% of the nonunion is described by most authors. Leg length inequallity, rotational and angular deformities can be corrected at the same time. During the period 1973-1995, we performed valgization osteotomy according to Pauwels in 66 patients of, 18-72 years old (mean 49.5 years). 24 (37%) of our patients died 4 months to 24 years (mean: 9.5 years) after the operation. Union of the femoral neck was achieved in 58 (88%) of the 66 patients; union of the osteotomy in 65 patients (99%). A good or excellent result was achieved in 62% (23 uneventful and 13 with healed, necrosis/arthrosis without need for further treatment) of our patients. However, the method has its limits. We feel if there is too little bone stock inside the femoral head, a valgization osteotomy does not give good result. The radiographic signs of avascular necrosis in patients over 30 years of age is considered a contraindication for an osteotomy. However our results show that it is worthwhile trying to save the joint of young patients even in case of a segmental collapse. In the race between revascularization and collapse, often revascularization is the winner. We deliberately give nature its chance and don't rely on the result of bleeding from drill holes in the head, nuclear scans and other methods to estimate vascularity. A secondary total hip replacement if necessary because of avascular necrosis or osteoarthritis is considerably postponed; and better milieu for hip replacement can be achieved by the development of sclerotic bone in the subchondral areas of the acetabulum and femoral head. Between 65 and 80 years of age, a total hip replacement is probably the best option for fit patients. We treat fresh femoral neck fractures with a hemiarthroplasty in patients over the biological age of 80 years. Logically the same choice will be made for patients with a nonunion. During the period 1973-1995 we performed hemiarthroplasty (*n* = 34) in patient with low general condition. Their mean age was 79 years. The average survival in these patients was less than three years and that explains probably the low late complication rate: in this group. Total hip replacement was performed in 37 younger patients with a mean age of 69 years. They were not considered for a valgization osteotomy because of age being over 70 years, severe osteoporosis or a total collapse of the femoral head. In this group, we observed one aseptic cup revision and two extractions of the prosthesis because of a deep infection.

## INTRODUCTION

**Figure 1:**
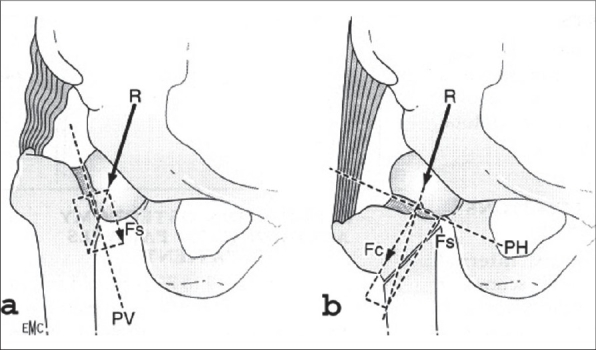
Principles of the valgization osteotomy designed by Pauwels. By taking out a lateral wedge, a steep fracture plane (PV) can be redirected into an almost horizontal one (PH). Mechanical analysis of the resultant force (R), acting on the nonunion, shows how in the preoperative situation shearing forces (Fs) dominate the fracture scene. After valgization R is almost parallel to the compression arm (Fc) of the parallelogram. R no longer displaces the fragments, but compresses them

Nonunion and avascular necrosis of the femoral head or a combination of both is the main complication following fractures of the femoral neck. In spite of improved operative techniques, nonunion is still reported in 10-20% of cases.^1^ The reason is a combination of unfavorable biomechanical and vascular conditions caused by the fracture itself, ignoring general contraindications, poor reduction and inadequate internal fixation. Usually there is shortening in the fracture, which limits the indication for simple refixation, the least radical operation.^1^ Pauwels classified the femoral neck fractures based on their mechanical behavior. As a logical consequence of his theories, he designed the abduction osteotomy at the intertrochanteric level [Figure 1], for the treatment of nonunions which converts shearing forces into compression.^2^ Nowadays in cases of nonunions of the femoral neck, the surgeon is tempted to perform prosthetic replacement of the hip; more so if there is also evidence of avascular necrosis of the head of femur. This provides rapid pain relief and allows early mobilization. However, long-term results of hip arthroplasties, especially in younger people and in the presence of osteopenia, are not always as expected; and a less radical approach is worth considering.

The combination of femoral neck nonunion and suspected avascular necrosis of the femoral head is no contraindication to a valgization osteotomy. Nonunion and osteotomy heal in these cases without any problem.^3^ Even in a partial collapse of the femoral head, good results can be achieved by abduction osteotomies in young patients, delaying a total hip replacement (THR) or hip fusion for many years. THR as a salvage procedure after unstable internal fixation or nonunion of the femoral neck is reserved for the elderly-patient group and patients with a severely deformed femoral head to avoid the complications of a valgus intertrochanteric osteotomy, such as recurrent nonunion, avascular necrosis and osteoarthritis of the hip joint.

From 1973 until 1995, we treated 143 patients operatively for a nonunion of the femoral neck in the Academical Medical Centre in Amsterdam. The three treatment modalities we used were refixation (*n* = 6), valgization osteotomy (*n* = 66) and prosthetic replacement (*n* = 71), which will now be discussed.

### 1. Refixation

**Figure 2:**
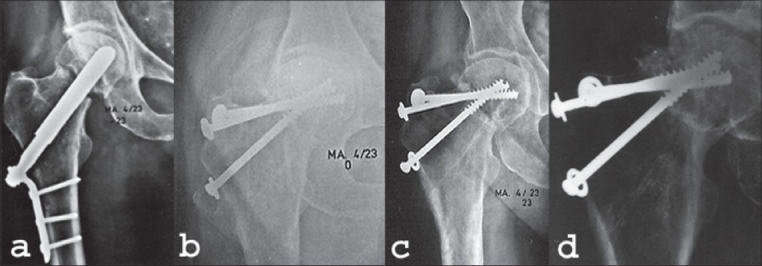
Refixation of a delayed union of the femoral neck in a 57-year-old woman. (a) Two months after Böhler nail shows distraction of the fracture. (b) postoperative X-ray after refixation with cancellous lag screws, acting as a tension band. (c) Five months after refixation. Fracture has healed. (d) followup at 4.5 years after refixation shows avascular necrosis of the head of the femur. The patient did not need a total hip arthroplasty until 14 years after refixation

Sometimes delayed primary internal fixation after failed nonoperative treatment of an impacted femoral neck fracture or refixation after failed internal fixation is possible without an osteotomy. This is especially a treatment option if leg length discrepancy is small or absent. This phenomenon is mainly seen after Pauwels type 2 fractures [Figure 2]. Fixation is according to the Pauwels type of the fracture, performed with screws (Pauwels 2) or an angled blade plate or dynamic hip screw (DHS) (Pauwels 3).

The fracture, shown in [Figure 2], was complicated by the development of avascular necrosis. This phenomenon can apparently not be avoided by this hardly radical operation. The risk of avascular necrosis is probably determined at the time of the primary fracture. An additional factor could be the unstable internal fixation, which works against revascularization of the femoral head.

### 2. Valgization osteotomy

Our strategy is to preserve the hip joint, not only of our younger patients but also in the active middle-aged group. Of course, the method has its limits. Severe incongruence of the femoral head leads to early osteoarthritis. Excavation of the head by moving hardware in unstable internal fixations offers too little chance for the nonunion to heal.

## SURGICAL PROCEDURE

### Preoperative planning

**Figure 3:**
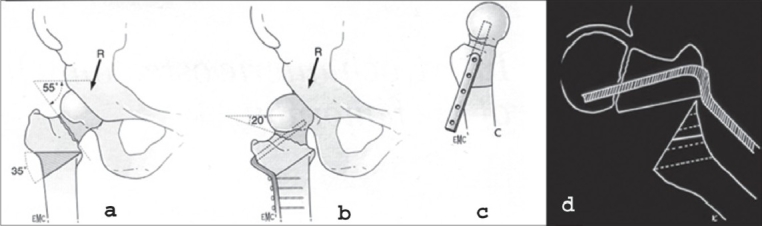
Preoperative drawing of a valgization osteotomy for femoral neck nonunion. (a) The inclination of the nonunion is the angle between the perpendicular to the femoral shaft and the fracture line. (b) Result of the preoperative planning. (c) Retrotorsion of more than 15° should be corrected and the position of the plate in the lateral view has to be included in the preoperative planning. (d) Loss of leg length is usual in femoral neck nonunions

The preoperative planning is performed by drawings from the plain X-rays. The amount of abduction is calculated on the anteroposterior (AP) view [Figure 3a,b].

If the osteotomy [Figure 3d] cannot compensate for the loss of leg length, a partial wedge should be taken in order to gain leg length. One of the reasons why we prefer the intertrochanteric osteotomy to the subtrochanteric osteotomy is the length of the osteotomy line in the former. Therefore, the contact area is big enough, even after taking out a partial wedge. Theoretically the ideal postoperative inclination angle to eliminate all shearing forces in the nonunion is approximately 20°. However, there is a limit to the degree of valgization, given by the range of adduction, that can be reached in the hip joint. Exceeding the permissible range of valgization introduces an abduction contracture. Clinical assessment of the correction possibility can be documented by taking an AP X-ray in maximal adduction, but the real range of adduction is greater than what clinical examination would suggest. Often the correction angle has to be evaluated during the operative procedure and falls between the optimal calculated angle and the maximal clinical adduction possibility. Severe flexion-extension-angulation deformity in the nonunion is rare. However, it has to be corrected in a way similar to a slipped epiphysis [Figure 3c]. Far more frequent is the rotational (external) deformity, which has to be evaluated clinically. Femoral neck nonunions are usually atrophic.

### Implants

The ideal implant for the valgization osteotomy is the AO 120° fixed angled blade plate. An alternative is the 95° condylar plate which can be bended to any desired angle, creating a shape similar to the 120° plate. Preoperatively, the use of an image identifier is recommended.

### Surgical standard procedure

**Figure 4:**
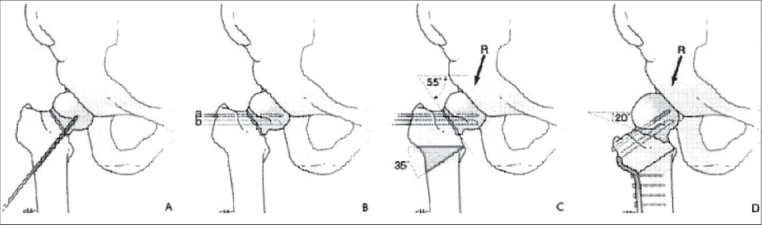
Standard technique of a Pauwels valgization osteotomy. (a) An extrosseous K-wire over the anterior side of the femoral neck marks the anteversion of the neck. (b) Parallel in the lateral plane to the K-wire in (a) a second intraosseous K-wire (a) is introduced, respecting the calculated valgization. (c) The seating chisel is introduced exactly parallel to the second wire and as caudally in the femoral head as possible. Image intensifier control in both directions and osteotomy is marked. Note: In order to avoid undue displacement of the nonunion during introduction of the seating chisel a cannulated cancellous screw can be driven home over K-wire a. (d) Lateral wedge is removed and valgization osteotomy is closed with well seated 120° angle blade plate

The intertrochanteric valgization osteotomy is demonstrated in a typical case [Figure 4] without significant angulation in the lateral view. The patient is in supine position on a standard operating table. A modified Watson-Jones anterolateral approach is advocated. Arthrotomy of the hip joint by excision of the anterior capsule is optional; it facilitates inspection of the femoral head during introduction of the seating chisel and the blade of the angled blade plate.

Two Kirschner wires are introduced in the sagittal plane for rotation control, one in the tip of the major trochanter and another distally from the future plate position. These wires should be inserted in a parallel way in absence of a rotational deformity. Usually the leg is in external rotation. The corresponding angle between the K-wires marks the angle over which the future distal fragment has to be internally rotated after the osteotomy. The K-wires should be parallel by then. The remaining steps of the operative procedure are described in the legends to Figure 4.

The proximal osteotomy is performed 2 cm distally to the seating chisel. A smaller amount of bone between the blade of the plate and the osteotomy increases the risk of break-out of the blade. In our example, a wedge of 35° is taken from the distal fragment. There are different surgical techniques of wedge resection: complete, partial and step by step. The standard procedure is the partial wedge resection in stages allowing several osteotomy reductions using the seating chisel as a handle to check the clinical adduction possibility. After removal of the seating chisel, the blade of the chosen 120°-angled blade plate is introduced. The blade is pushed forward by hand parallel to the guide wire [Figure 4a–d]. The osteotomy is reduced by closing the wedge and applying the plate to the femoral shaft with a clamp. This action leads automatically to compression of the osteotomy. Whenever there is too much abduction in the hip joint at the end of the procedure, never change the position of the seating chisel. The seating chisel if manipulated will become loose. In such situation the angle of the plate to the new situation is to be adapted. Fixation of the plate to the femur using the asymmetric dynamic compression (DC) holes for additional compression concludes the procedure. Finally, check the residual range of adduction and the parallelism of the K-wires for rotational control.

Note: A neutral position between abduction and adduction should be aimed at. Even an abduction contracture up to 10° can be accepted; but under these circumstances, it is advisable to install a traction-suspension for 1 or 2 weeks.

### Pitfalls

**Figure 5:**
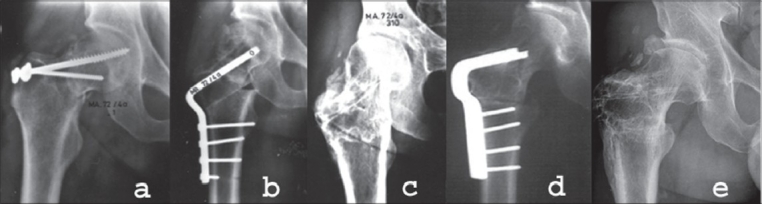
Secondary varization because of osteotomy in too much valgus: (a) AP x-ray in a 26 years male shows 8 weeks old fixation for femoral neck fracture. (b) Pauwels valgization osteotomy was performed in too much of valgus. (c) Six years followup x-ray of the same patient after implant removal shows signs of osteoarthrosis hip and patient was symptomatic. (d) Postoperative x-ray shows intertrochanteric varus osteotomy. (e) Ten years after last operation. The patient has mild pain and patient can walk with cane for 45 minutes, Harris hip score 79
- b. **Too little bone stock in femoral head:** If the bone stock in femoral head is less then the chances of implant cutout increases [Figure 6].

- **Too much valgization:** It is obvious that hip biomechanics is changed by the valgization. Severe valgus of the femoral neck is a mechanically unfavorable condition. Enormous forces are acting on the hip joint and overload-osteoarthritis may develop. Extreme valgization may lead to a subluxation of the femoral head, especially in dysplastic hips. Therefore, the younger patients have to be checked at regular intervals. If the hip gets symptomatic, a secondary or varization might be indicated [Figure 5].

**Figure 6:**
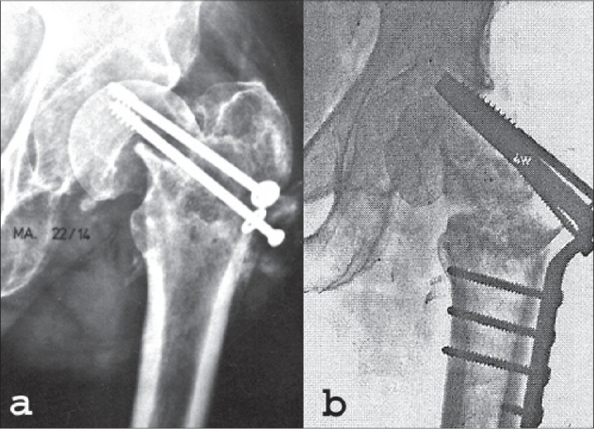
Wrong indication for valgization osteotomy. (a) Male, 50 years. Seven months after internal fixation. Nonunion. The screw tips have continuously been turning around in the femoral head and have scraped away the cancellous bone. An empty eggshell-like situation resulted, but was not appreciated. (b) Four weeks after a valgization osteotomy; implants have not enough hold in this excavated femoral head. Cut-out of the blade and screw. In this case a primary prosthetic replacement would have been a better choice than a valgization osteotomy
- **Splitting-up of the femoral head during introduction of the seating chisel:** In young patients, the cancellous bone in the femoral head can be very hard. The risk of splitting the femoral head during introduction of the seating chisel can be reduced by pre-drilling with a 4.5 mm drill bit. Opening of the hip joint provides a direct view of the femoral head and helps to avoid this complication.
- **Wrong length of the blade of the plate and wrong introduction:** Correct length of the blade of the plate is very important. The blade should be chosen long enough to offer sufficient hold in the femoral head. If the plate is too short, secondary instability may occur [Figure 7a,b].

**Figure 7:**
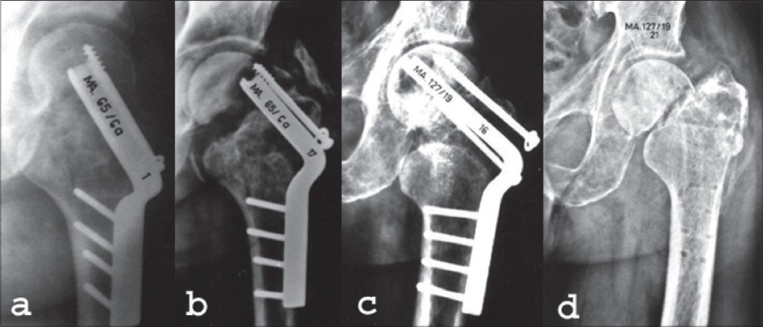
Consequences of a wrongly chosen length of the blade of the plate. (a) and (b) The blade is too short, has insufficient grip in the femoral head and breaks out. (c) and (d) The blade is too long, the femoral head cannot sink over the plate to come in firm contact with the femoral neck

On the other hand, if the blade is too long, there is a risk of nonunion, caused by distraction [Figure 7c,d]. The length of the blade must be at least 1 cm less than the distance over which the seating chisel has been driven in. The proximal fragment should have the possibility to glide over the blade towards the distal fragment.

The introduction of the blade is a critical part of the procedure. In order to prevent a false route of the blade, take care that the exposure of the introduction point is excellent. Introduce the plate by hand and not mounted on the device that the manufacturer has provided for it.

## COMPLICATIONS AND RESULTS

During the period 1973-1995 valgization osteotomy according to Pauwels was performed in 66 patients, 18-72 years old (mean 49.5 years). There was no hospital mortality. During the observation period, 24 (37%) of our patients died 4 months to 24 years (mean: 9.5 years) after the operation due to unrelated reasons. One patient developed postoperative hematoma, which was debrided. The angled blade plate had to be exchanged in four cases because the blade penetrated into the hip joint. Union of the femoral neck was achieved in 58 (88%) of the 66 patients, union of the osteotomy in 65 patients (99%). The long-term results are listed in Table 1.

**Table 1:** Results of 66 valgization osteotomies for femoral neck nonunion

	No. of cases	Treatment	Follow-up	HHS	Satisfaction
Persistent nonunion	8	THR - 6			
		Refixation - 2	15 years	70	
		Union, necrosis - 1	11 years	82	
		Persistent, nonunion - 1			
Healed nonunion uneventful	23	No	> 10 years	80-100 average 95	100%
Healed nonunion avascular necrosis/arthrosis, no further treatment	13	No	10-26 years	62-67, average 73	94%
Healed nonunion with avascular necrosis/arthrosis, addition treatment	22	THR - 21			
		Hip fusion - 1			
		Average interval osteotomy re-operation = 9 years			

HHS - Harris hip score

**Figure 8:**
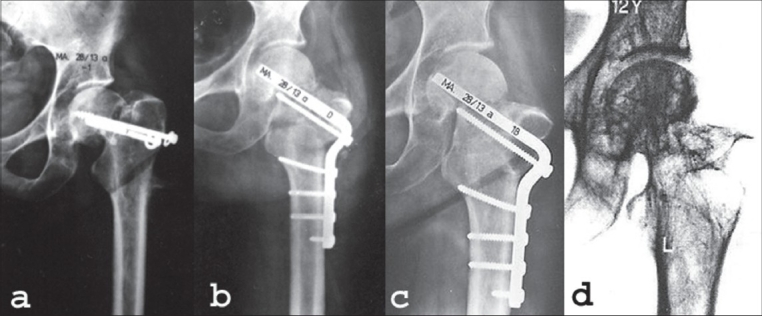
(a) Female, 43 years, femoral neck nonunion with very steep fracture line. Gross valgization is needed. (b) The Y-shaped osteotomy allows a high valgization degree and the medial displacement leads to direct support of the femoral head by the calcar femoris. (c) Five months postoperative. The aspect of the femoral head is avascular/sclerotic. Note the screw through the plate in the proximal fragment, which improves the hold of the plate in the proximal fragment. (d) At 12 years after valgization the femoral head shows the irregular pattern, resulting from an intensive revascularization process. However, there is no deformity of the head; the hip joint is congruent

A good or excellent result was achieved in 62%: uneventful healing in 23 cases and healing with avascular necrosis/arthrosis without need for further treatment in 13 cases of our patients. Our results showed that deformity caused by avascular necrosis can be avoided by timely revascularization [Figure 8].

**Figure 9:**
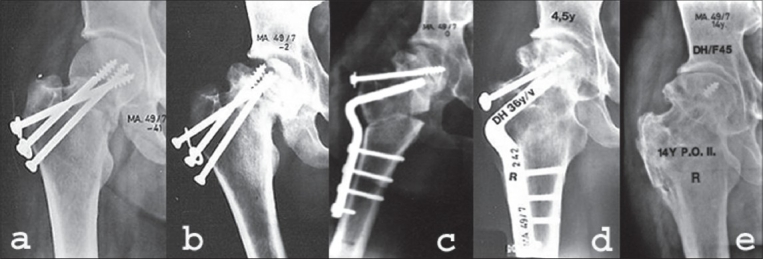
Female, 30 years. (a) Three months after screw fixation of a displaced femoral neck fracture shows healing fracture. The patient did not report for followup. (b) Two years after the primary fracture the patient was readmitted after a fall. She now seems to have a pathological fracture in the necrotic femoral head. (c) Postoperative X-ray after open reduction of the fracture was performed. The fracture was stabilized only by a screw and valgization osteotomy. (d) Healed osteotomy and fracture, 4.5 years postoperative. (e) Fourteen years after the osteotomy. There is little trochanteric pain, the patient walks one hour without any support

Of special interest is the group of five patients in whom the deformity of the femoral head was already visible at the operation. Undisturbed healing of the nonunion was observed in each of these five cases. The significance of the delay of prosthetic replacement which a Pauwels osteotomy can achieve in young patients is illustrated in [Figure 9]. Under stable conditions, revascularization of the femoral head (fragment) is possible but the final result is not predictable. In 21 patients out of the group of 35 patients with a healed nonunion and avascular necrosis/arthrosis, a total hip replacement was performed, 0.5-27 years (mean nine years) after the osteotomy. A total hip replacement was considerably postponed until better bone stock was gained.

## DISCUSSION

Usually nonunions of the femoral neck are grossly displaced by shortening and rotation. If not, a simple (re)fixation of the initial fracture without complementary osteotomy can be successful, as long as the original fracture line corresponds to the Pauwels types 1 and 2. However, in our small group of six patients with such a procedure, three of them developed serious avascular necrosis.

Hou reports a small series of neglected fractures with shortening up to 5 cm. He was able to cure these nonunions with a pedicled autologous bone graft and restore leg length in four of his five patients.^4^ The use of these grafts has been popularized by Meyers.^5^ The initially reported success has not been reproduced in large series and the procedure has been considered unreliable.^6^

The valgization osteotomy, designed by Pauwels, represents a masterly mechanical concept, with which not only healing of the nonunion and osteotomy can be achieved but also leg length discrepancy, rotational and angular deformity can be corrected at the same time. Osteotomies on subtrochanteric level^7^,^8^ are less capable to correct the inclination of the fracture line adequately; to restore leg length and secondary THR gets more difficult. Finally the cancellous bone of the intertrochanteric region offers better healing qualities than the cortical bone at the subtrochanteric level.^9^ We do not consider that preoperative traction^7^,^10^ is necessary. Restoration of leg length can be achieved by valgization and varying the wedge, taken from the distal fragment. We never required a wedge of more than 55°. A 120°-plate as standard implant is satisfactory in case requiring 50-60° correction.

The question whether a valgization osteotomy interferes with the vascularity of the femoral head should be addressed. Calandruccio concluded that the vascular damage at the time of the fracture decides whether or not necrosis will develop.^11^ This conclusion is supported by the not-yet-published results of internal fixation of fresh femoral neck fractures in our institution. In a series of 228 patients who could be followed for 3-19 years, the frequency of avascular necrosis was the highest (57%) in the group of patients whose fracture did heal after internal fixation, but in a position other than the immediate or early postoperative position. Temporary instability apparently has a greater impact on the vascularity than does an osteotomy and introduction of an angled blade plate (45% avascular necrosis). We therefore consider the femoral neck nonunion like Pauwels a mechanical problem and the addition of pedicled or unpedicled autologous bone graft^7^,^12^ superfluous. Several authors who use the same technique as we described reported good early results in adults^1^,^6^,^8^,^13^–^16^ and children.^17^ Together, these authors reported on 118 cases, which means that our series of 66 patients is by far the largest published ever [Table 2].

**Table 2:** Valgization osteotomy for femoral neck nonunion (review of the larger series)

Author	Year of publication	Number	% Union	Age limits
Weber and Cech^13^	1973	36	100% (?)	“Younger”
Lies and Scheuer^14^	1983	17	88%	<60 years
Walcher and Wiesinger^15^	1983	13	100% (?)	<40 years
Wentzensen and Weller^16^	1983	7	100%	<60 years
Ballmer *et al.*^9^	1990	17	88%	<60 years
Anglen^6^	1997	13	100%	<60 years
Mathews and Cabanela^1^	2004	15	80%	±70 years
Magu *et al.*^17^	2007	10	100%	Children

Walcher^15^ considered radiographic signs of avascular necrosis in patients over 30 years of age as a contra-indication for an osteotomy. Our results show that it is worthwhile trying to save the joint of young patients even in case of a segmental collapse [Figure 9]. In the competition between revascularization and collapse, often revascularization will be the winner. We deliberately give nature its chance and don't rely on the result of bleeding from drill holes in the head^18^ nuclear scans and other methods to measure vascularity.

**Figure 10:**
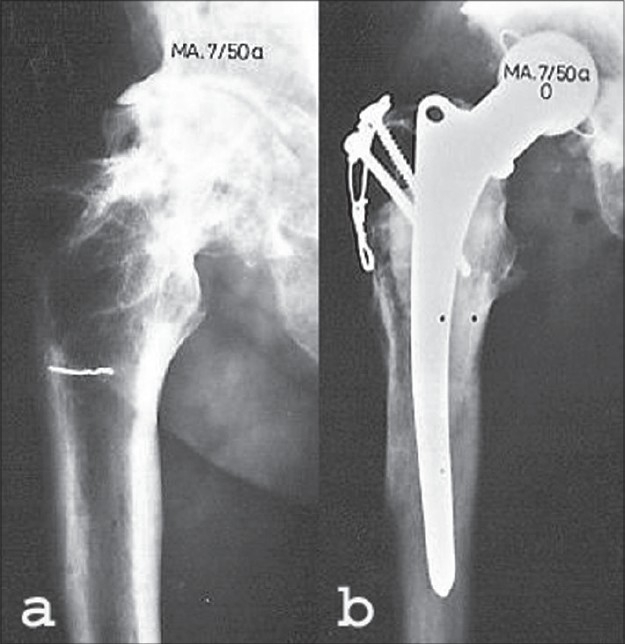
(a) Female 71 years, 16 years after valgization osteotomy; osteoarthritis secondary to avascular necrosis. The sclerotic changes in the femoral head and acetabulum improve the chances for a good longterm result of THR. (b) Postoperative X-ray after cemented THR

A total hip replacement is considerably postponed and better conditions for hip replacement can be achieved by the development of sclerotic bone in the subchondral areas of the acetabulum and femoral head [Figure 10].

The results of the valgization osteotomy related to age are listed in Table 3. It is obvious that patients in their sixties should not be excluded from having a joint-saving operation. In four of our patients, the proximal femoral fragment was brought in too much valgus. The postoperative AP-Garden-Index was 180°. This mechanically unfavorable situation caused in three older patients a late osteoarthritis, followed by total hip replacement. A younger patient had an intertrochanteric varus osteotomy 9 years after the Pauwels osteotomy [Figure 5], with a good result.

**Table 3:** Results of valgization osteotomy related to age

Age group (mean age)	Nonunion/necrosis	THR% (interval between osteotomy and reoperation in years)	Mean FU/HHS (no reoperation group)
<40 years (28)	0%/54%	23%/12 years	11 years/88
40-49 years	25%/54%	15%/7 years	9 years/79
50-59 years	4%/54%	44%/9 years	11 years/93
>60 years (65)	33%/25%	25%/2 years	5 years/89

### 3. Prosthetic replacement

It is our strategy to treat fresh femoral neck fractures with a hemiarthroplasty (HA) in patients over the biological age of 70 years. Logically the same choice will be made for patients with a nonunion. During the period 1973-1995, we performed an HA 34 times in the least vital group of 34 patients. Their mean age was 79 years. The average survival in these patients was less than 3 years and that explains probably the low late complication rate with only one aseptic loosening and one deep infection with chronic fistula.

Total hip replacement (THR) was performed in 37 younger patients with a mean age of 69 years. They were not considered for a valgization osteotomy because of age being over 70 years, severe osteoporosis or a total collapse of the femoral head. In this group, we observed one aseptic cup revision and two extractions of the prosthesis because of a deep infection.

Literature on the treatment of femoral neck nonunion is scarce. Reports on HA or THR for nonunion are not frequently published either. However, these reports are, without exception, in favor of THR as the treatment for femoral neck nonunion. Mehlhoff^19^ described the results of THR for femoral neck and trochanteric nonunion. He observed postoperative dislocations exclusively in the trochanteric group. The results in the femoral neck group were comparable to those of THR for fresh fractures. Johnson^20^ compared HA and THR and focused on postoperative dislocations. Dislocations were mainly seen after HA Franzen^21^ reported THR in 84 patients with femoral neck fracture: nonunion did better than reported results of THR for acute femoral neck fracture. Secondary THR after failed internal fixation had an outcome similar to that of primary THR, except for a higher incidence of mechanical failure of the prosthesis in older patients. Other reports confirm the 1.5-2.5 times higher risk of secondary THR compared to primary THR for osteoarthritis.^1^,^22^,^23^ Therefore, these authors plead for the choice of joint-saving procedures (refixation, valgization osteotomy) in younger patients, if the local situation permits such a choice (absence of complete collapse) and if the surgical skill is available. However, approximately 8,000 femoral neck fractures per year occur in the Netherlands. About 25% of these patients are under 70 years of age and should therefore be treated with internal fixation. According to the findings of Lu-Yao^24^ at least 300 nonunions should be produced in 1 year. We are afraid that the majority of these 300 nonunions in our country will be treated with an arthroplasty instead of a Pauwels osteotomy. Some missionary work has still to be done!

## References

[1] Mathews V, Cabanela ME (2004). Femoral neck nonunion treatment. Clin Orthop Relat Res.

[2] Pauwels F (1935). The femoral neck fracture a mechanical problem leaflet issue to Zeitschr f Chir Orthop, Band 63.

[3] Marti RK, Schüller HM, Raaymakers EL (1989). Intertrochanteric osteotomy for nonunion of the femoral neck. J Bone Joint Surg Br.

[4] Hou SM, Hang YS, Liu TK (1993). Ununited femoral neck fractures by open reduction and vascularized iliac bone graft. Clin Orthop Relat Res.

[5] Meyers MH (1980). The role of posterior bone grafts (muscle pedicle) in femoral neck fracture. Clin Orthop Relat Res.

[6] Anglen JO (1997). Intertrochanteric osteotomy for failed internal fixation of femoral neck fracture. Clin Orthop Relat Res.

[7] Huang CH (1986). Treatment of neglected femoral neck fractures in young adults. Clin Orthop Relat Res.

[8] Zinghi GF, Specchia L, Ruggieri N, Galli G (1985). The role of osteotomy in the treatment of pseudarthrosis of the neck of the femur in younger patients. Ital J Orthop Traumatol.

[9] Ballmer FT, Ballmer PM, Baumgartel F, Ganz R, Mast JW (1990). Pauwels osteotomy for nonunions of the femoral neck. Orthop Clin North Am.

[10] Stewart MJ, Wells RE (1956). Osteotomy and osteotomy combined with bone grafting for non-union following fracture of the femoral neck. J Bone Joint Surg Am.

[11] Calandruccio RA, Anderson WE (1980). Post-fracture avascular necrosis of the femoral head: Correlation of experimental and clinical studies. Clin Orthop Relat Res.

[12] Schwetlick G, Weber U, Klingmuller V (1989). The femoral neck pseudarthrosis following medial femoral neck fracture. An indication for the vascularized pedicled iliac crest transplant. Unfallchirurg.

[13] Weber BG, Cech O, Weber BG, Cech O (1973). Schenkelhals-pseudarthrose. Pseudarthro sen: Pathophysiology, Biomechanics, therapy, results.

[14] Lies A, Scheuer I (1983). Pseudo-arthrosis of the neck of the femur in adults: Pathogenesis, therapy and results. Unfallheilunde.

[15] Walcher K, Wiesinger H (1983). Pauwels valgization osteotomy or alloplasty in pseudarthrosis of the femur neck. Aktuel Traumatol.

[16] Wentzensen A, Weller S (1983). Pseudarthrosis as a complication of femoral neck fracture. Aktuel Traumatol.

[17] Magu NK, Singh R, Sharma AK, Ummat V (2007). Modified Pauwel's intertrochanteric osteotomy in neglected femoral neck fractures in children: A report of 10 cases followed for a minimum of 5 years. J Orthop Trauma.

[18] Gill TJ, Sledge JB, Ekkernkamp A, Ganz R (1998). Intraoperative assessment of femoral head vascularity after femoral neck fracture. J Orthop Trauma.

[19] Mehlhoff Th, Landon GC, Tullos HS (1991). Total hip arthroplasty following failed fixation of hip fractures. Clin Orthop Relat Res.

[20] Johnsson R, Bendjelloul H, Ekelund L, Persson BM, Lidgren L (1984). Comparison between hemiarthroplasty and total hip replacement following failure of nailed femoral neck fractures, focused on dislocations. Arch Orthop Trauma Surg.

[21] Franzen H, Nilsson LT, Strömqvist B, Johnsson R, Herrlin K (1990). Secondary total hip replacement after fracture of the femoral neck. J Bone Joint Surg Br.

[22] Skeide BI, Lie SA, Havelin LI, Engesaeter LB (1996). Total hip arthroplasty after femoral neck fractures: Results from the national registry on joint prostheses. Tidsskr Nor Laegeforen.

[23] Jackson M, Learmonth ID (2002). The treatment of nonunion after intracapsular fracture of the proximal femur. Clin Orthop Relat Res.

[24] Lu-Yao GL, Keller RB, Littenberg B, Wennberg JE (1994). Outcomes after displaced fractures of the femoral neck. J Bone Joint Surg Am.

